# Risk of Postpartum Dental Caries: Survival Analysis of Black/African American and White Women in Appalachia

**DOI:** 10.1089/whr.2023.0056

**Published:** 2024-02-15

**Authors:** Morgan Byrd, Elyse Davis, Freida Blostein, Deesha Bhaumik, John R. Shaffer, Daniel W. McNeil, Mary L. Marazita, Betsy Foxman

**Affiliations:** ^1^ Department of Epidemiology, Center of Molecular and Clinical Epidemiology of Infectious Diseases, School of Public Health, University of Michigan, Ann Arbor, Michigan, USA.; ^2^ Department of Oral and Craniofacial Sciences, Center for Craniofacial and Dental Genetics, School of Dental Medicine, University of Pittsburgh, Pittsburgh, Pennsylvania, USA.; ^3^ Department of Human Genetics, School of Public Health, University of Pittsburgh, Pennsylvania, Pennsylvania, USA.; ^4^ Department of Community Dentistry and Behavioral Science, College of Dentistry, University of Florida, Gainesville, Florida, USA.; ^5^ Clinical Translational Science, School of Medicine, University of Pittsburgh, Pittsburgh, Pennsylvania, USA.

**Keywords:** cohort studies, dental public health, women's health, epidemiology

## Abstract

**Background:**

Pregnancy is associated with increased risk of caries, but the extent this increase extends into the postpartum period is poorly understood.

**Study Objective:**

Describe the epidemiology of dental decay in the postpartum period among Black/African American and White American women and explore associations with potentially modifiable risk factors.

**Materials and Methods:**

We analyzed data from 1,131 Black/African American and White women participating in Center for Oral Health Research in Appalachia cohorts. Women were enrolled during the first two trimesters of pregnancy. Calibrated dental professionals completed dental examinations at the prenatal enrollment visit, and 2-month, 1-year, 2-year, and 3-year postpartum visits.

**Results:**

Between the prenatal visit and 2-month visit, the incidence of decayed, missing, and filled teeth (DMFT) increase was 6.92/100 person-months, compared to 3.6/100 person-months between the 2-month and 1-year visit. In a multivariate Cox proportional hazard regression predicting incidence of caries up to 3-years postpartum, being younger, having less than college education, a household income <$50,000, smoking cigarettes, a DMFT >0, a very poor or poor Oral hygiene Rating Index, lower salivary pH at enrollment, or frequently drinking 100% juice increased the hazard of new dental caries. Adjusting for race/ethnic group did not affect the direction or magnitude of observed associations.

**Conclusions:**

The strong associations of prior DMFT and Oral Rating Index with occurrence of new dental caries postpartum suggests that targeting young women for interventions to improve oral health may be more valuable for reducing caries incidence during pregnancy and in the postpartum period than targeting women only during pregnancy.

## Introduction

Pregnant women are at higher risk for oral disease, including dental decay, than nonpregnant women of the same age,^1–3^ and important racial and ethnic differences exist in pre- and postnatal dental care and in prevalence of dental problems.^4^ Whether postpartum women are also at higher risk for dental decay is poorly studied.^5^ One hypothesized mechanism for the increase of caries throughout pregnancy is that decreasing salivary pH enhances growth of acidophilic cariogenic bacteria.^6^ This hypothesis is supported by results from an Indian study of 50 pregnant women in which lower salivary pH levels persisted until 6 weeks postpartum.^1^ In addition to salivary pH, other caries risk factors (including potentially modifiable factors such as diet and access to dental care) might contribute to postpartum increases in decayed, missing, and filled teeth (DMFT).

We previously conducted an analysis of 879 White women participating in Center for Oral Health Research in Appalachia cohort 2 (COHRA2). In the previous analysis, we tested for associations between diet and increases in DMFT count between 2-months and 6-years postpartum.^7^ Using a modified Poisson model predicting an increase in the number of DMFT up to 6-years postpartum, we found that greater frequency of drinking 100% fruit juice increased risk of new dental caries by ∼10%, and consumption of vegetables decreased risk by a similar percentage, after controlling for important confounders, including geographic location, age, dental and medical insurance, family income, and education level.

However, the previous analysis was limited to White women and did not include the early (<2-month) postpartum period when many important physiological and sociobehavioral changes occur.^8^ Further, it did not include an investigation of the effect of salivary pH, which may change throughout the postpartum period and may explain an increase in the risk of dental decay.^1^

We found only three studies that assessed changes in women's risk of caries between pregnancy and postpartum, all focused on the early postpartum period. The study by Kamate et al. measured the oral health of 50 women during each trimester of their first pregnancy and at 6 weeks postpartum and oral health at a single time point for 50 age-matched nonpregnant women.^1^ Among the pregnant women, median DMFT increased from two to three by the third trimester; among nonpregnant controls, median DMFT was two. A Chinese study collected unstimulated saliva samples from 24 healthy women during each trimester of pregnancy and again at 6 weeks postpartum.^2^ The authors observed a pathogenic shift toward microbes associated with gingivitis and caries during pregnancy that reverted back to a healthy microbiome by 6 weeks postpartum.

A limitation of the Indian and Chinese studies is the short follow-up period, and small sample sizes. By contrast, in an Iranian study among women with a history of dental decay, women assigned to an educational intervention (*n* = 236) and controls (*n* = 200) had similar modest increases in mean DMFT between the second/third trimesters and their 24-months postpartum follow-up (intervention: 10.96 to 11.01; controls: 9.44 to 9.58).^3^

Given the paucity of data on risk of caries during the early postpartum period, we conducted a preliminary analysis to assess the incidence of a new carious lesions among COHRA2 participants between the first or second trimester prenatal visit and the 2-month postpartum visit. We observed a rate of 10.0 new carious teeth/100 person-months. This rate seemed surprisingly high compared to reports from nonpregnant adults: a Swedish study of 982 nontobacco using young men and women followed for 3-years observed a mean increase in the average number of decayed and filled surfaces (DFS) of 1 (∼2.8/100 person-months).^9^

Believing our observation required further investigation, here we estimate time to incidence and incidence rate of DMFT increase between pregnancy and 2-months, 1-year, 2-years, and 3-years postpartum in the COHRA White (COHRA2) and Black/African American (COHRASmile) cohorts. Our objective is to further describe the epidemiology of dental decay in the postpartum period among Black/African American and White women and explore associations with potentially modifiable risk factors.

## Materials and Methods

### Study population

**FIG. 1.**
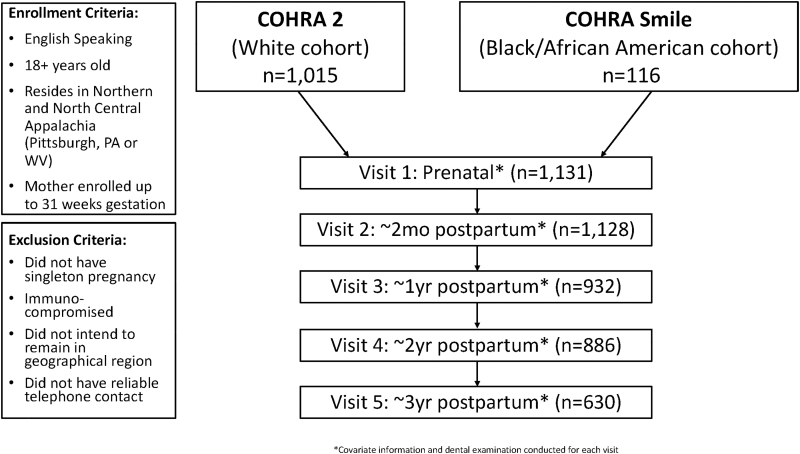
Flowchart of two cohorts of 1,131 mothers participating in COHRA2 & COHRA Smile enrolled between November 2011 and March 2019, with visit frequency, and criteria for enrollment and exclusion. COHRA2, Center for Oral Health Research in Appalachia Cohort 2.

COHRA2 is a mother-child pair prospective study of English-speaking pregnant White women aged 18 years and older residing in Northern and North Central Appalachia, specifically in Pittsburgh, Pennsylvania, and throughout the state of West Virginia.^10^ A Black/African American cohort was implemented in October 2017 (COHRASmile) using the same protocol as COHRA2. The current study included 1,015 participants from COHRA2 continuously enrolled between November 2011 and February 2017 (followed through May 2020) and 116 participants from COHRASmile enrolled between October 2017 and March 2019 (followed through August 2021) (Fig. 1). Mothers were enrolled up to 31 weeks' gestation and followed postpartum at 2-months, 6-months (Pennsylvania only), 12-months, and yearly thereafter for up to 6-years postpartum.

Women were excluded if they did not have a singleton pregnancy, were immunocompromised, did not think they would remain in the general geographic region for the duration of the study, or if they did not have reliable telephone contact.^10^ This analysis included 1,131 women enrolled between November 2011 and March 2019 who completed at least one postpartum visit in the first 3-years of follow-up. Among those included, 1,128 completed a 2-month postpartum visit, 932 completed a 1-year postpartum visit, 886 completed a 2-year postpartum visit, and 630 completed a 3-year postpartum visit; these numbers decrease partly due to loss to follow-up and partly because participants had not reached the 2-year or 3-year visit mark. The study protocol was approved by the Institutional Review Boards at the University of Pittsburgh and West Virginia University.^10^

### Data collection

Mothers gave written consent at enrollment and provided detailed demographic, dental and medical history, and oral health care information. In addition, mothers were queried in 6 monthly telephone interviews regarding diet, oral health care, demographic, and dental and medical information that might change during follow-up. The interviews were conducted by the University of Pittsburgh Center for Social and Urban Research (UCSUR).^10^ At enrollment, a sample of household water was collected and tested for fluoride. For the 11 cases where 2 water samples were provided, the fluoride levels were averaged.

At enrollment and each following in-person visit, participants were examined by calibrated, licensed dental professionals (dentist or dental hygienist).^10^ The complete dental examination included measurement of salivary pH, assessment of oral health using the Oral Rating Index (ORI), and caries assessment following the PhenX Toolkit Dental Caries Experience Prevalence Protocol.^10–12^ ORI is a visual measure of gingival health and oral hygiene.^11^ Normal gingival condition and no detectable plaque or calculus is rated as excellent (+2), normal gingival condition with a small amount of plaque and calculus as good (+1), localized gingival inflammation and small amount of plaque and calculus as questionable (0), gingival information and notable amount of plaque and calculus as poor (−1), and gingival inflammation and large amount of plaque and calculus accumulation as very poor (−2).

The reader is referred to the 2022 article by Kedokteran et al., which includes photographs corresponding to the scores.^13^ The tooth codes were modified and included both DMFT and surface scores. Note that for this study, we used DMFT as our outcome and did not include white spots, that is, the beginning stages of tooth enamel demineralization which are capable of remineralization.

We calculated the change in DMFT between the date of each in-person visit and enrollment for each study participant. As our outcome, we used the incidence rate of DMFT increase (number of first identified DMFT increase divided by time of follow-up in person-months) considering a month of 30 days. The timing of each participant's visits varied; therefore, the person-time by visit date was more accurate than using the target timing of follow-up visit. For a graphical display using a Kaplan–Meier survival curve, the delivery date was considered Day 0 and time was added cumulatively for each subsequent visit date. For rate of DMFT increase, the prenatal enrollment visit was considered Day 0 to capture person-time contributed from the prenatal enrollment visit to the first postpartum visit.

A decrease in DMFT is not likely, as DMFT are not reversible, so any negative changes identified between visits were evaluated and recoded as no change (*n* = 97) assuming a potential miscount during the previous dental examination. Miscounts were most often observed among participants with higher DMFT scores (52% with a score of 8 or higher, 69% with a score of 6 or higher).

Site, race, education, ORI, and household income were measured as categorical variables. Frequency of vegetable and 100% juice consumption were also categorical, with responses of never, every few days, once a day, and several times a day. Dental insurance, dental visits, floss, smoking, and fluoride in toothpaste were all dichotomous (yes/no) variables. Floss frequency and smoking frequency were evaluated as categorical variables for those who indicated that they did floss or smoke. As pH is measured on a log scale, salivary pH was log-transformed for analysis as a continuous variable. Mother's age at enrollment, DMFT at enrollment, number of prior pregnancies, and household fluoride were also analyzed as continuous variables. However, for presentation in the rate analysis (Table 1), continuous variables were categorized.

**Table 1.** Mean Decayed, Missing, and Filled Teeth at Time of Enrollment and Incidence of Decayed, Missing, and Filled Teeth Increase/100 Person-Months by Mother's Fixed Covariates^*^

	Mean DMFT (SD)		Incidence of DMFT during interval/100 person-months^a^			
		Prenatal enrollment visit	Prenatal to 2-month visit	2-month to 1-year visit	1- to 2-year visit	2- to 3-year visit
Characteristic	***N***	(***n*** = 1131)	(***n*** = 1128)	***(n*** = 932)	(***n*** = 886)	(***n*** = 630)
Total	1131	6.75 (5.51)	6.92	3.6	1.65	1.7
Site						
Pittsburgh, PA	630	6.67 (5.47)	5.78	3.57	1.22	1.76
West Virginia	501	6.84 (5.56)	8.25	3.63	2.17	1.60
DMFT at enrollment						
0	123	0.00 (0)	1.64	2.35	1.36	0.66
1–2	75	1.00 (0)	4.27	2.56	2.41	1.00
3–7	494	4.35 (1.74)	7.38	4.45	1.72	1.96
8+	439	12.31 (4.35)	8.23	3.23	1.52	1.90
Mother's age at enrollment						
18–25	328	6.78 (5.77)	9.07	5.64	3.35	2.14
26–29	289	6.15 (5.40)	5.77	3.91	1.77	1.91
30–32	246	6.55 (5.29)	5.33	2.18	1.25	1.04
33+	268	7.53 (5.42)	6.81	2.45	0.67	1.90
All ages	1131	6.75 (5.51)	6.92	3.60	1.65	1.70
Race						
White	1006	6.72 (5.52)	7.16	3.66	1.6	1.71
Black/African American	111	6.59 (4.69)	3.71	3.22	3.59	NA
Multiracial^b^	9	9.67 (9.50)	18.03	0	7.92	NA
Other race	3	9.67 (10.69)	14.49	NA	NA	NA
Missing	2	13.50 (7.78)	0	0	0	NA
Number of prior pregnancies						
0	419	5.82 (5.01)	6.94	4.15	1.41	1.48
1	323	6.59 (5.42)	6.00	2.06	1.82	1.79
2+	389	7.88 (5.88)	7.66	4.56	1.72	1.88
Education						
<High school	52	10.35 (6.62)	13.06	13.56	2.44	2.02
High school diploma	199	8.23 (6.40)	10.26	4.19	4.09	3.49
Some college	245	6.83 (5.44)	8.40	4.49	1.69	2.04
College degree	366	6.23 (5.22)	4.79	2.69	1.39	1.35
Master's or Doctorate	268	5.54 (4.33)	4.49	2.89	1.13	1.56
Unk/missing	1	19.00 (NA)	0	0	0	NA
Household fluoride in water (ppm)^c^						
0.00–0.69	335	6.60 (4.90)	6.59	4.73	0.97	1.93
0.70–1.20	686	6.65 (5.61)	7.08	2.52	1.72	1.61
1.21–1.84	54	8.43 (5.33)	7.82	4.40	2.28	1.38
Unk/missing	56	7.14 (7.22)	5.93	10.02	5.18	1.98

^*^ One thousand one hundred thirty-one mothers participating in COHRA2 and COHRA Smile, enrolled between November 2011 and March 2019.

^a^ Those without a DMFT measure during the visit were excluded from the table counts.

^b^ Multiracial includes the following responses: White/Native American or Alaskan Native, African American/Native American or Alaskan Native, African American/Other Race, White/African American/Native American or Alaskan Native, and White/African American/Asian.

^c^ The United States Public Health Service recommends a fluoride level of 0.7 ppm for community water systems.^13^

DMFT, decayed, missing, and filled teeth; SD, standard deviation.

### Statistical analysis

Our analysis distinguished between fixed variables collected at enrollment: site, baseline DMFT, mother's age, race, number of prior pregnancies, education, and household fluoride in water (Table 1) and time-varying covariates collected via UCSUR telephone interviews: dental insurance coverage, dental visits (assessed at the end of each interval), flossing habits, smoking habits, ORI, salivary pH, household income, fluoride in toothpaste, frequency of 100% fruit juice consumption, and frequency of vegetable consumption. Fruit juice and vegetable consumption was assessed using a 7-day food frequency questionnaire with predefined categories (Table 2). Our previous analysis of dietary variables identified increased risk of new dental caries between 2-months and 6-years postpartum associated with drinking 100% juice, and a decreased risk of new dental caries with eating vegetables.^7^

**Table 2.** Mean Decayed, Missing, and Filled Teeth at Time of Enrollment and Incidence of First Decayed, Missing, and Filled Teeth Increase/100 Person-Months by Mother's Time-Varying Covariates by Postpartum Visit^*^^,^^a^

	Prenatal visit	Prenatal to 2-month visit		2-month to 1-year visit		1- to 2-year visit		2- to 3-year visit	
Characteristic	(***n*** = 1131)	(***n*** = 1128)		(***n*** = 932)		(***n*** = 886)		(***n*** = 630)	
	Mean (SD)	***n***	Incidence/100 person-months	***n***	Incidence/100 person-months	***n***	Incidence/100 person-months	***n***	Incidence/100 person-months
	6.75		6.92		3.6		1.65		1.7
Household income									
<$25,000	8.77 (6.57)	281	9.82	310	5.08	270	2.80	235	2.73
$25,000-$49,999	6.39 (5.70)	203	7.89	202	3.81	210	2.01	213	2.1
$50,000-$74,999	6.22 (4.59)	173	5.03	154	2.13	140	1.14	140	1.16
$75,000+	5.88 (4.69)	469	5.41	425	3.20	408	1.05	365	1.52
Missing/Unk	7.40 (5.59)	5	0	40	4.53	31	7.94	26	0
Dental insurance									
Yes	6.69 (5.45)	799	6.25	799	3.34	741	1.22	724	1.66
No	6.87 (5.71)	298	8.49	282	3.94	278	2.53	222	1.60
Missing/Unk	7.00 (5.23)	34	8.13	50	7.50	40	7.00	33	6.84
Dental visit^b^									
Yes	6.73 (5.51)	1096	7.63	541	4.04	492	1.47	274	1.62
No	7.17 (5.69)	30	6.27	481	3.00	456	1.60	173	1.54
Missing/Unk	7.40 (5.59)	5	8.35	37	4.65	31	5.18	247	1.92
Floss									
Yes	6.62 (5.16)	821	6.66	781	3.09	774	1.33	734	1.65
No	7.09 (6.34)	305	7.70	312	5.07	255	2.38	218	2.08
Missing/Unk	7.40 (5.59)	5	0	38	4.88	30	7.94	27	0
Floss frequency^c^									
Never-<1/day	6.19 (4.96)	493	6.15	476	2.97	461	1.37	440	1.67
1/day	6.96 (5.35)	244	6.08	240	3.46	244	1.27	230	1.61
2/day	7.17 (5.25)	63	10.17	43	2.32	51	0	49	1.39
3+/day	10.95 (5.33)	21	14.60	22	2.98	18	4.22	15	2.71
Smoking									
Yes	9.59 (6.65)	163	9.60	229	5.06	245	2.58	219	2.77
No	6.25 (5.15)	952	6.15	865	3.29	785	1.34	735	1.57
Unknown	7.44 (4.87)	16	20.82	37	5.32	29	7.94	25	0
Smoking frequency^c^									
Rarely	8.88 (6.01)	8	6.82	20	2.75	28	0.64	20	3.02
<1/2 pack	9.28 (6.52)	115	10.10	164	4.08	161	2.26	129	1.62
>1/2 pack	10.71 (8.07)	24	7.81	35	9.71	49	5.63	54	4.32
1+ pack	10.60 (4.56)	5	22.51	10	13.32	5	8.28	10	8.92
ORI									
Very poor (−2)	12.45 (8.22)	55	10.60	64	5.84	53	3.42	48	3.31
Poor (−1)	8.02 (5.95)	250	8.24	258	3.57	191	2.58	177	2.85
Questionable (0)	5.70 (4.03)	10	14.55	5	5.92	8	0	4	0
Good (+1)	6.30 (4.90)	582	6.04	569	3.63	470	1.32	469	1.51
Excellent (+2)	5 (4.03)	232	4.26	231	2.01	209	1.10	187	1.11
Missing/Unk	28 (0)	2	0	4	0	128	0	94	NaN
Salivary pH									
Highly acidic (<6)	7.91 (5.98)	46	3.23	19	1.16	23	4.91	32	0
Moderately acidic (6–6.6)	6.95 (5.28)	481	5.77	336	4.19	395	1.81	467	1.44
Healthy (>6.6)	5.68 (4.98)	141	5.43	407	2.17	345	0.99	367	1.82
Missing/Unk	6.75 (5.81)	463	8.87	369	5.16	296	2.42	113	4.89
Fluoride toothpaste									
Yes	6.63 (5.42)	872	6.71	903	3.77	898	1.62	837	1.56
No	7.65 (6.37)	89	8.17	67	0.68	57	1.72	70	3.44
Missing/Unk	6.89 (5.47)	170	7.30	161	3.96	104	1.84	72	1.62
100% juice									
Never	6.55 (5.47)	434	6.17	547	2.71	610	1.37	597	1.70
Every few days	6.27 (5.04)	371	6.64	318	4.37	253	1.73	212	1.43
Once/day	7.18 (6.00)	216	7.40	147	3.85	108	1.93	96	2.60
Several times/day	8.33 (5.94)	104	10.09	80	6.15	59	2.43	49	1.67
Missing/Unk	7.50 (5.01)	6	0	39	4.90	29	7.94	25	0
Vegetables									
Never	6.34 (5.19)	59	7.91	49	1.31	46	1.88	46	4.00
Every few days	7.50 (6.41)	220	7.99	196	2.82	172	2.52	161	1.72
Once/day	6.65 (5.63)	431	6.37	458	3.92	380	1.17	348	1.53
Several times/day	6.50 (4.85)	416	6.83	392	3.70	432	1.52	401	1.69
Missing/Unk	7.40 (5.59)	5	0	36	5.92	29	7.94	23	0

^*^ One thousand one hundred thirty-one Mothers Participating in COHRA2 and COHRA Smile, Enrolled Between November 2011 and March 2019.

^a^ Those without a DMFT measure during the visit were excluded from table counts. The covariate categorizations are based on the measure at time 1 and each column reflects the rate of DMFT during that applicable visit (time 2). The prenatal enrollment average DMFT by covariates is based on the covariate at prenatal visit as we do not have the time-varying covariates leading up to that period (see [Materials and Methods]).

^b^ The covariate measure is reflective of the prior visit, reflecting the time-period leading up to the dental examination except for the dentist visit question. This question was asked specifically of the time-period leading up to the examination and is the only covariate analyzed at time 2 (see [Materials and Methods]). Therefore, the n for this covariate will not align with the n of the other covariates.

^c^ Not all flossers and smokers answered the frequency question; therefore, only those that answered were included.

We evaluated incidence of DMFT increase between interval start versus interval end for the following intervals: prenatal visit to 2-months postpartum, 2-months to 1-year postpartum, 1-year to 2-year postpartum, and 2-year to 3-year postpartum. Once women had an increase in DMFT, they were no longer considered at risk and were removed from the analysis of subsequent time periods. Women who were lost to follow-up were censored. This approach resulted in all covariate responses predictive of dental caries risk reflecting the time-period leading up to the visit where DMFT was measured. ORI was evaluated at interval start and interval end as ORI may impact DMFT and vice versa. As a sensitivity analysis, we compared model results, including ORI at interval start to ORI at interval end; both measures had similar associations with dental caries risk.

**FIG. 2.**
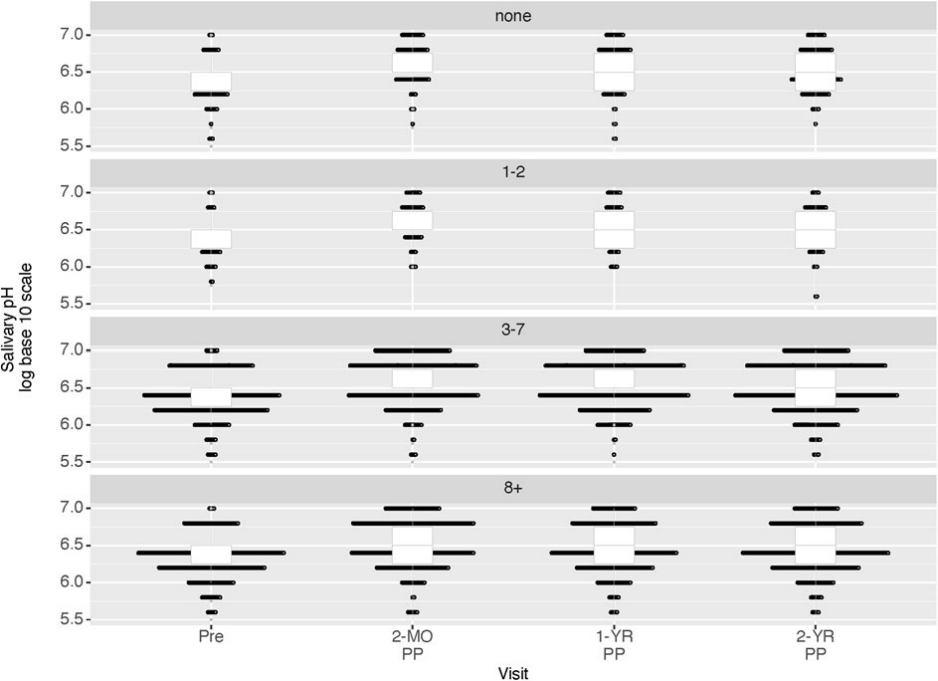
Dot plot identifying individual salivary pH measures by visit and baseline DMFT of 1,131 mothers participating in Center for Oral Health Research in Appalachia (COHRA) Cohort 2 and COHRA Smile, enrolled between November 2011 and March 2019. DMFT, decayed, missing, and filled teeth.

**FIG. 3.**
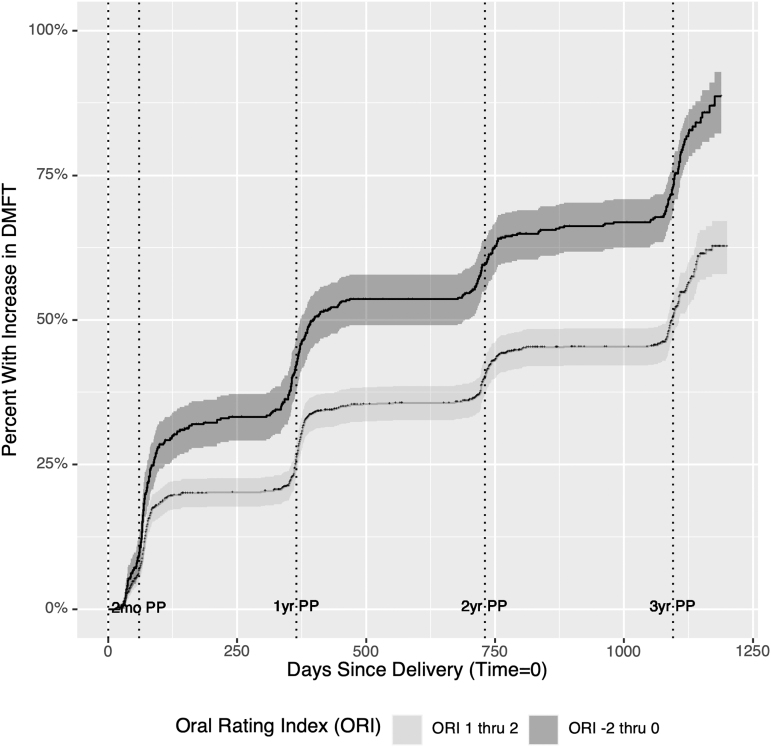
Inverted Kaplan-Meier curve showing percent with increase in DMFT in days with good (ORI 1–2) and poor (ORI −2 to 0) ORI. One thousand one hundred thirty-one moths participating in the Center for Oral Health Research in Appalachia (COHRA) Cohort and COHRA Smile, enrolled between November 2011 and March 2019. ORI, Oral Rating Index.

Salivary pH was compared by baseline DMFT and across all visits. Although salivary pH is a continuous variable, due to the limitations of pH measurements, there was clustering of distinct values observed. As a result, the changes in salivary pH are shown with a dot plot to identify individual observations, treating this measure as a discrete variable for visualization (Fig. 2). We visualized time to DMFT increase postpartum using an inverted Kaplan–Meier survival curve by ORI category (Fig. 3). To estimate hazard ratios (HRs) and 95% confidence intervals adjusted for covariates, we conducted a multivariate Cox proportional hazard regression analysis. Both fixed and time-varying covariates were considered for inclusion. Because of the high level of missing data for salivary pH, we fit two separate models, one including and one excluding salivary pH.

Covariates for both models were selected for inclusion based on prior evidence of association with dental caries in the literature, for example, tobacco use, oral health, and access to dental care,^14^ or a statistically significant (*p* < 0.05) univariate association with an increase in DMFT. If no association with a variable was identified in the univariate or a confounder in stratified analyses, the variable was not included in model fitting.

The final models were constructed using baseline prenatal DMFT score (DMFT = 0 vs. >0, as increasing levels of DMFT, categorized as 1–2, 3–7, 8+, all had similar HRs), mother's age at enrollment, ORI, log-transformed salivary pH (complete case only), frequency of 100% juice consumption, smoking status, household income (< vs. ≥$50,000), and education ( vs. ≥college degree). As including vegetable consumption had no impact on the HRs, it was not included in the final model, and *p*-values of <0.05 were considered significant. All analyses were conducted using RStudio Version 1.4.1106.

## Results

### Characteristics of the study population

Among the 1,131 participating mothers, the average baseline DMFT was 6.7 (standard deviation [SD] 5.51) (min 0, max 28). Average DMFT scores were higher at enrollment for participants who were older, had multiple pregnancies, less than a high school education, a household income <$25,000, had no dental insurance, smoked, had a low ORI (range from −2 to 2; −2 is very poor and +2 excellent), and lower salivary pH (more acidic) (Tables 1 and 2). Although those who reported any flossing had lower DMFT compared to those who reported no flossing, among the individuals who reported flossing, those who flossed 3+/day had a higher DMFT than those who flossed less than once, once, or twice a day, suggesting that greater flossing was a response to higher DMFT. There was a positive association between increasing number of cigarettes smoked and average DMFT.

Salivary pH was not measured for 40% of participants at enrollment, and ∼30% of participants at each follow-up visit (Table 2). However, among those with salivary pH measurements, salivary acidity decreased (higher pH) over follow-up, regardless of DMFT at enrollment with pH clustering between 6 and 7 (Fig. 2). The mean (SD) salivary pH at the prenatal visit was 6.37 (0.37) and increased to 6.66 (0.41) at the 2-month visit and stayed at a higher pH than at enrollment thereafter [6.60 (0.42), 6.58 (0.42), 6.54 (0.43) for the 1-year, 2-year and 3-year visits, respectively] (one-way repeated measures ANOVA *p*-value <0.001).

### Incidence of DMFT increase postpartum

Between the prenatal visit and 2-month visit, the incidence of DMFT increase was 6.92/100 person-months, compared to 3.6/100 person-months between the 2-month and 1-year visit (Table 1). The incidence of DMFT increase postpartum was greatest for the prenatal to 2-month interval for all fixed covariates except for women with a DMFT of 0 at enrollment and those with less than a high school education (Table 1) and for all time-varying covariates except smoking frequency >1/2 pack (Table 2).

Incidence of DMFT increase of 8/100 person-months or greater between enrollment and the 2-month visit was observed for participants who lived in West Virginia, had a DMFT of eight or more, were 18 to 25 years of age, did not have a college degree, had a household income <$50,000, had no dental insurance, flossed two or more times a day, smoked cigarettes, had a poor or very poor ORI, did not use fluoride toothpaste, or drank 100% juice several times a day. The incidence between enrollment and the 2-month visit was much higher among White than Black/African American participants (7.16 vs. 3.71/100 person-months), however, the incidence was relatively stable across all follow-up visits for Black/African American participants, but decreased for White participants across subsequent visits.

Twenty six percent of participants had an increase in DMFT between enrollment and their 2-month visit and almost half of participants (49%) had an increase in DMFT by the 3-year visit. The average time from enrollment to increase in DMFT was 73.6 days (SD 37.2) for those who experienced an increase at the 2-month postpartum visit and 376.3 (SD 35.2), 745.1 (SD 50.2), and 1110.1 (SD 59.4) days for those whose increase was at the 1-year, 2-year, and 3-year postpartum visits, respectively.

As women with a poor or very poor ORI had high rates of increase in DMFT, we illustrate the cumulative incidence of DMFT increase following delivery among those with an ORI of 0 or below (questionable, poor, very poor) compared to 1 or 2 (good, excellent) at each visit using an inverted Kaplan-Meier survival curve. The slope of increase was lower at every interval for those with good or excellent ORI (Fig. 3) but was still greatest between enrollment and 2-months postpartum.

### Factors associated with increases in DMFT

We fit a multivariate Cox proportional hazard regression model to estimate HRs adjusted for covariates. All covariates included in Tables 1 and 2 were considered during model building. Because of the high level of missingness of measures of salivary pH, we fit two models, one including salivary pH and one excluding salivary pH. We assessed possible interactions for smoking and income, smoking and salivary pH, income and education, and ORI and baseline DMFT. None of these interactions was statistically significant. In both models, the strongest predictors of increased risk of a new caries lesion in the 3-year postpartum was having a DMFT >0. Being younger age, having an excellent or good ORI score, having a college degree, and being in the Black/African American cohort decreased risk.

As salivary pH increased—becoming more alkaline—risk of a new caries also decreased. Salivary pH appears to explain the association with 100% juice consumption, as when pH is included the association with juice decreases and is no longer statistically significant (Table 3). In both models, nonsmokers of cigarettes had lower hazards, but they did not reach statistical significance.

**Table 3.** Results of Multivariate Cox Proportional Hazard Regression Analysis of First Incidence of New Caries During the 3-Years of Follow-Up, Including and Excluding Salivary pH^*^

Analysis including salivary pH (complete case) (*n* = 517)			
Model components	HR	95% CI	***p***
Baseline/prenatal DMFT 1+^a^	1.88	1.28–2.76	0.001
Mother's age at examination (years)^b^	0.98	0.96–1.00	0.042
Excellent or good ORI^a,c^	0.64	0.52–0.78	1.75e-5
Log transformed salivary pH (lower pH is more acidic)^b^	0.22	0.06–0.83	0.026
100% juice consumption^a,d^	1.04	0.94–1.14	0.48
Non-cigarette smoker^a^	0.87	0.69–1.09	0.23
Household income >$50,000^a^	0.96	0.78–1.19	0.72
Education college degree or greater^a^	0.82	0.65–1.03	0.09
Race (African American)^e^	0.70	0.49–0.99	0.045

Analysis excluding salivary pH (*n* = 812)			
Baseline/prenatal DMFT 1+^a^	2.04	1.50–2.78	6.19e-6
Mother's age at exam (years)^b^	0.98	0.97–0.99	0.007
Excellent or good ORI^a,c^	0.70	0.59–0.82	1.35e-5
100% juice consumption^a,d^	1.12	1.04–1.20	0.003
Non-cigarette smoker^a^	0.88	0.74–1.05	0.16
Household income >$50,000^a^	0.99	0.84–1.17	0.92
Education college degree or greater^a^	0.76	0.63–0.90	0.002
Black/African American^e^	0.56	0.40–0.79	0.0007

^*^ Adult Participants in COHRA2 and COHRA Smile enrolled between November 2011 and March 2019.

^a^ Reference groups of baseline DMFT score of 0, poor ORI score (−2 to 0), never frequency of 100% juice consumption, smoker, household income <$50,000, and education of <college degree.

^b^ Variables are continuous variables and do not have reference group.

^c^ The ORI ranges from very poor (−2) to excellent (+2).

^d^ Responses to 7-day food frequency recall: never, every few days, once a day, several times a day.

^e^ Mixed and other races were not included in the model due to small sample size.

CI, confidence interval; HR, hazard ratio; ORI, Oral Rating Index.

## Discussion

This prospective study of time to increase in DMFT among Black/African American and White Appalachian women during the postpartum period adds greatly to the sparse literature on the epidemiology of dental caries postpartum. We identified the first 2-months postpartum as the period of greatest incidence of DMFT increase (6.92/100 person-months): almost twice as high as the rate observed between the 2-month and 1-year visit (3.60 per 100/person-months). This early increase and subsequent fall was mirrored in continued acidity of salivary pH during early postpartum period and subsequent decrease in acidity previously reported.^1,2^ Other factors associated with shorter time to increase in DMFT, such as younger age, lower education and socioeconomic status, cigarette smoking, juice consumption, and previous poor oral health outcomes, are consistent with previous reports.

Although Black/African American women had a longer time to increase in DMFT, adjusting for self-identified race made no difference in the direction or significance of the associations of other variables and time to increase in DMFT. These results show that increased risk of dental decay associated with pregnancy extends into the early postpartum period and the strong influence of socioeconomic factors on risk of dental decay, highlighting the need for continuity of dental care before, during, and following birth.

We observed a lower 2-month incidence of DMFT increase among Black/African American women (3.71 per 100/person-months) than White women (7.16 per 100/person-months). However, the incidence between 2-months and 1-year was similar among Black/African American and White women (3.22 vs. 3.66 per 100/person-months), and higher among Black/African American than White women between 1 and 2 years postpartum (3.59 vs. 1.6 per 100/person-months). These differences likely stem from the small number of Black/African American participants (*n* = 116) and selection due to loss-to-follow-up. However, given that the Black/African American participants were enrolled 6-years after initiating enrollment of the White cohort, changes in policies or programs might also explain part of these differences.

The observed changes in salivary pH between pregnancy and the postpartum period support the hypothesis that changes to the acidity of the oral cavity during the partum and postpartum period increase risk of dental caries. Salivary pH can be altered by numerous mechanisms, including diet, oral hygiene habits, changes in oral microbiome composition, and physiological changes and therefore salivary pH could mediate associations between modifiable risk factors and caries incidence in the postpartum period.^6^ Including salivary pH in multivariable models did not substantially change HRs for important modifiable risk factors such as smoking and diet in our analysis, although the *p*-values of these associations were decreased likely due to decreased sample size and subsequent decrease in power. Thus, it is possible that smoking and diet influence caries incidence through other pathways than salivary pH.

Several factors associated with incidence of DMFT increase in postpartum are also associated with oral health disparities: low income, lower education, and cigarette smoking are associated with poorer ORI and greater DMFT. ORI captures gingival health and oral hygiene; poor oral hygiene is also associated with dental caries. DMFT and ORI capture past use of dental care. In nonpregnant populations, tobacco use is associated with changes in the oral microbiome leading to increased risk of gingivitis and dental caries.^5,15^ We observed in an earlier analysis of the COHRA2 cohort during the postpartum period that an increase in DMFT in the prior follow-up interval was strongly associated with increased risk in DMFT in the subsequent interval.^7^

In the current study, a shorter time to an increase in DMFT was associated with a baseline DMFT >0 (compared to DMFT = 0) but time to increase did not change with greater baseline DMFTs suggesting a possible threshold effect. Making dental care more accessible and affordable to women with lower incomes would help improve ORI and reduce DMFT.

Appalachia currently—and historically—has significant overall health and oral health disparities, including lack of access to affordable dental care. Although we did not observe racial disparities in caries incidence among this cohort of Black/African American Appalachian women, the literature suggests they occur.^4^ Black/African American women are less likely to have a teeth cleaning before and during pregnancy and the postpartum period even though they are more likely to have a dental problem.^7^ Potential selection and follow-up biases and the small number of Black/African American participants might have obscured racial disparities.

The dental literature has focused primarily on the effect of maternal oral health and behaviors on offspring oral health rather than maternal health when considering the partum and postpartum period.^6,16^ Thus, there is relatively little in the literature about the incidence and risk factors for dental caries during pregnancy and especially the postpartum periods, and we found no studies among Black/African Americans. Determining if incidence is indeed elevated requires data from a nonpregnant control group, ideally of the same age range followed for a similar time-period. We found no such studies among U.S. populations for comparison. Although not ideal as an external control, results from a Finnish study of 13,564 males and 255 females provide some insight.

This study measured the prevalence of dental caries among young healthy adults over a 6-month period. There was a modest increase in DMFT among the males (4.1 to 4.3 or ∼3.3 per 100 person-months) and no increase among the females (3.9 at each time point or 0 per 100 person-months).^17^ While there are substantial differences in study population and access to care, this does imply that among Appalachian postpartum women, the observed increase in DMFT between the first or second trimester of pregnancy and 2-months postpartum, and between 2-month and 1-year postpartum was higher than might be expected.

A Swedish prospective study compared risk of increase in number of DFS among tobacco and nontobacco users over a 3-year period. Among the 982 participants, the number of DFS increased by 0.9 (SD 1.6) or ∼2.5/100 person-months among nontobacco users and 1.4 (SD 2.4) or ∼3.9/100 person-months for tobacco users.^9^ Our study counted DMFT and excluded white spots (*i.e*., DFS counts all decayed and filled lesions so one tooth may have multiple decayed or filled surfaces, and potentially reversible demineralization). Even so, the rates in the Swedish study are lower than observed in the 1-year postpartum among smokers and nonsmokers in our postpartum cohort.

Our study has several strengths, including the large sample size, the longitudinal design that included annual dental examinations by calibrated, licensed dental professionals, and collection of detailed information on sociodemographics, oral health habits, and medical and dental history. We analyzed data from both Black/African American and White women. We used a conservative measure, DMFT, which excludes white spots; therefore, it is unlikely that the high incidence in the early postpartum period is attributable to an increase in transient lesions.

Further, DMFT counts a tooth as decayed or filled regardless of the number decayed or filled surfaces on the tooth, so we likely underestimated caries incidence. We also considered time-varying changes in flossing and tobacco use—habits associated with increased risk of dental decay. COHRA participants are from a region of the United States at high risk of dental decay and other oral health and overall health problems; so, the focus is on a population that has considerable health disparities and inequities.^18^

However, our study has some limitations; restrictions of the salivary pH measurement resulted in distinct clustering, but this was considered through the evaluation of a complete case model and one excluding salivary pH. In addition, the dates of the enrollment periods differed between our White and Black/African American cohorts that could explain some of the differences observed between the two cohorts. Due to the small sample size of the Black/African American cohort, selection and follow-up biases may also have been present that obscured the racial disparities within our findings that literature suggest exist. There are also known oral health disparities among the Appalachian region; therefore, this may limit the generalizability of our findings to other regions.

## Conclusion

Physiologic changes in the mother—including changes in the oral microbiome—and lifestyle changes, such as smoking habits, that occur during pregnancy and postpartum likely contribute to increased dental decay in the postpartum period. Pregnant women are already encouraged to receive dental care while pregnant; our findings suggest that they should additionally be encouraged to receive dental care in the early postpartum period.^19^ However, given the strong associations of prior DMFT and ORI with caries incidence, focusing attention on improving oral health among young women would potentially do more to reduce caries incidence in the postpartum period.

## Abbreviations Used

CI: confidence interval
COHRA2: Center for Oral Health Research in Appalachia cohort 2
DMFT: decayed, missing, and filled teeth
DFS: decayed and filled surfaces
HR: hazard ratio
ORI: Oral Rating Index
SD: standard deviation
UCSUR: University of Pittsburgh Center for Social and Urban Research
